# A circRNA ceRNA network involved in cognitive dysfunction after chronic cerebral hypoperfusion

**DOI:** 10.18632/aging.205387

**Published:** 2024-01-16

**Authors:** Wan-Rong Jiang, Yong-Ming Zhou, Wei Wu, Li-Jie Yang, You Wu, Xin-Yuan Zhang, Zhao-Hui Yao

**Affiliations:** 1 Department of Geriatrics, Renmin Hospital of Wuhan University, Wuhan, China; 2 Henan Key Laboratory of Neurorestoratology, The First Affiliated Hospital of Xinxiang Medical University, Xinxiang, Henan, China; 3 Department of Clinical Laboratory, Renmin Hospital of Wuhan University, Wuhan, China

**Keywords:** chronic cerebral hypoperfusion, cognitive dysfunction, RNA sequencing, ceRNA

## Abstract

Chronic Cerebral Hypoperfusion (CCH) is associated with cognitive dysfunction, the underlying mechanisms of which remain elusive, hindering the development of effective therapeutic approaches. In this study, we employed an established CCH animal model to delve into neuropathological alterations like oxidative stress, inflammation, neurotransmitter synthesis deficits, and other morphological alterations. Our findings revealed that while the number of neurons remained unchanged, there was a significant reduction in neuronal fibers post-CCH, as evidenced by microtubule-associated protein 2 (MAP2) staining. Moreover, myelin basic protein (MBP) staining showed exacerbated demyelination of neuronal fibers. Furthermore, we observed increased neuroinflammation, proliferation, and activation of astrocytes and microglia, as well as synaptic loss and microglial-mediated synapse engulfment post-CCH. Utilizing RNA sequencing, differential expression analysis displayed alterations in both mRNAs and circRNAs. Following gene ontology (GO) and Kyoto Encyclopedia of Genes and Genomes (KEGG) enrichment analyses, both showed significant enrichment in immunological and inflammation-related terms and pathways. Importantly, the differentially expressed circular RNAs (DE circRNAs) exhibited a notable coexpression pattern with DE mRNAs. The ternary circRNA-miRNA-mRNA competing endogenous RNAs (ceRNA) network was constructed, and subsequent analysis reiterated the significance of neuroimmunological and neuroinflammatory dysfunction in CCH-induced neuropathological changes and cognitive dysfunction. This study underscores the potential role of circRNAs in these processes, suggesting them as promising therapeutic targets to mitigate the detrimental effects of CCH.

## INTRODUCTION

Chronic cerebral hypoperfusion (CCH) widely exists in many neurodegenerative diseases, which can progressively lead to cognitive impairment. CCH builds an important bridge between vascular cognitive impairment and dementia [1]. Because CCH is a reversible pathophysiological condition, there are intervenable opportunities and strategies to mitigate its detrimental effects on cognitive dysfunction. It is crucial to urgently attain a deep understanding of the underlying mechanisms of CCH’s effect on cognition, which will contribute to resolving the many neurodegenerative diseases and even design and develop effective therapeutic methods. CCH disrupts the blood supply to the brain resulting in glucose and oxygen hypometabolism [2], which can induce oxidative stress, inflammation, synthesis decrease of neurotransmitters, white matter lesions, hippocampal atrophy, and more [3]. Oxidative stress (OS) can impair neurons and nerve fibers, leading to apoptosis, pyroptosis, and ferroptosis. Meanwhile, OS can damage myelin sheath, nerve fibers, and mitochondria, leading to a cascade of inflammatory responses by activating astrocytes and microglia. Hence, it is one of the main injuries that neuroinflammation brings damage to neurons and its related protrusions. Therefore, figuring out the injury mechanisms of CCH-related neuroinflammation will contribute to resolving cognitive dysfunction. However, there is a lot of heterogeneity among CCH animal models, which generally contributes to the vastly different results regarding the mechanisms of CCH. The heterogeneity of CCH’s animal models consists of the uncertainty of lesion sites, variations in the extent of brain tissue damage, differences in animal model replication methods, and inconsistencies in the responsiveness and compensatory effects of individual animals to model replication. These factors eventually lead to experimental results with too much heterogeneity to truly understand the critical and comprehensive mechanisms of CCH’s effect on cognitive dysfunction. To address this predicament, repeatable and stable animal model production methods need to be developed. By comparing various animal model methods, it has been found that bilateral ligation of the common carotid artery with a specific time interval results in minimal brain injury and very high survival rates among experimental animals. Using this replication method, the animal model demonstrates relatively high stability, allowing for a reliable assessment of the extent of cognitive dysfunction and neuropathophysiological changes. These will provide a reliable basis to further analyze the underlying mechanisms.

CCH could induce multiple injury mechanisms in brains by regulating many expressions of inflammatory genes and related signaling pathways. The NRF2/ARE/NF-κB signaling pathway impairs cognition in rats through processes involving inflammation, oxidative stress, and ferroptosis following CCH [4]. Downregulation of sirt1/PGC-1α exacerbates mitochondrial damage and increases ROS production and inflammation in CCH models [5]. Drd2/Cryab/NF-κB pathway aggravates cognitive dysfunction through increasing inflammation and apoptosis [6]. These suggested that there are lots of signaling pathway alterations involving the inflammation and neuropathological changes after CCH. It was not difficult to imagine that there is a complex regulatory network for neuroinflammation after CCH. Generally, the single signaling pathway only reflects the one point, only the multiple pathways embody the one aspect. The regular study method makes it difficult to accomplish this task. As well as known, RNA sequencing can display many altered genes to be further analyzed for understanding the network alterations of many diseases [7]. Based on the trait of RNA sequencing, the underlying network should be explored after CCH. circRNA has been discovered in recent years and plays a sponge role in competing for the miRNAs with binding mRNAs to regulate the expression of mRNAs. Circular RNA circ-FoxO3 can improve blood-brain barrier damage in cerebral ischemia/reperfusion [8]. circHECTD1 can compete to bind microRNA-142 and inhibit miR142- activity to decrease astrocyte activation [9]. circRNA-Memo1 can inhibit the miRNA-17-5p/SOS1 pathway to aggravate cerebral hypoxia/reoxygenation injury [10]. Nevertheless, the all-round regulatory functions of circRNAs remain unresolved [11, 12], not to mention their intricate regulatory networks involving interactions with miRNAs and mRNAs. Recent study showed that whole transcriptome including circRNA and other small RNAs changed dramatically after CCH [11, 12]. circRIMS2 could ameliorate cognitive impairment after CCH through the circRIMS2/miR-186/BDNF axis [13]. circular RNA could reduce neuronal apoptosis after CCH via the circ_0000296/miR-194-5p/Runx3/Sirt1 axis [14]. These suggested that circRNA played important role in impairment after CCH. So, what role circRNA in whole plays after CCH also remains unclear. The RNA sequencing can detect the differentially expressed circRNAs, which can be predicted to regulate what miRNA is by matching the existing database. Therefore, the circRNA-miRNA-mRNA regulatory network will be possibly investigated to uncover the regulatory mechanisms through RNA sequencing. Hence, RNA sequencing and data analysis will contribute to understanding the neuroinflammation regulatory network by constructing the circRNA-miRNA-mRNA regulatory network after CCH. It can be imaged that the underlying regulatory mechanisms can be unveiled and the key targets can be identified for prevention and treatment of cognitive dysfunction through RNA sequencing analysis after CCH.

In the present study, the cognitive dysfunction after CCH and neuropathological changes in the CNS would be re-evaluated, including neurons, nerve fibers, myelin sheaths, and immune cells. Through RNA sequencing and bioinformatics methods, we explored the underlying regulatory mechanisms of neuropathological changes, particularly the regulatory roles of circRNAs in neuroinflammation through constructing the competing endogenous RNA network after CCH. These would contribute to understanding the mechanisms of cognitive dysfunction after CCH.

## MATERIALS AND METHODS

### Animals

**Figure 1:**
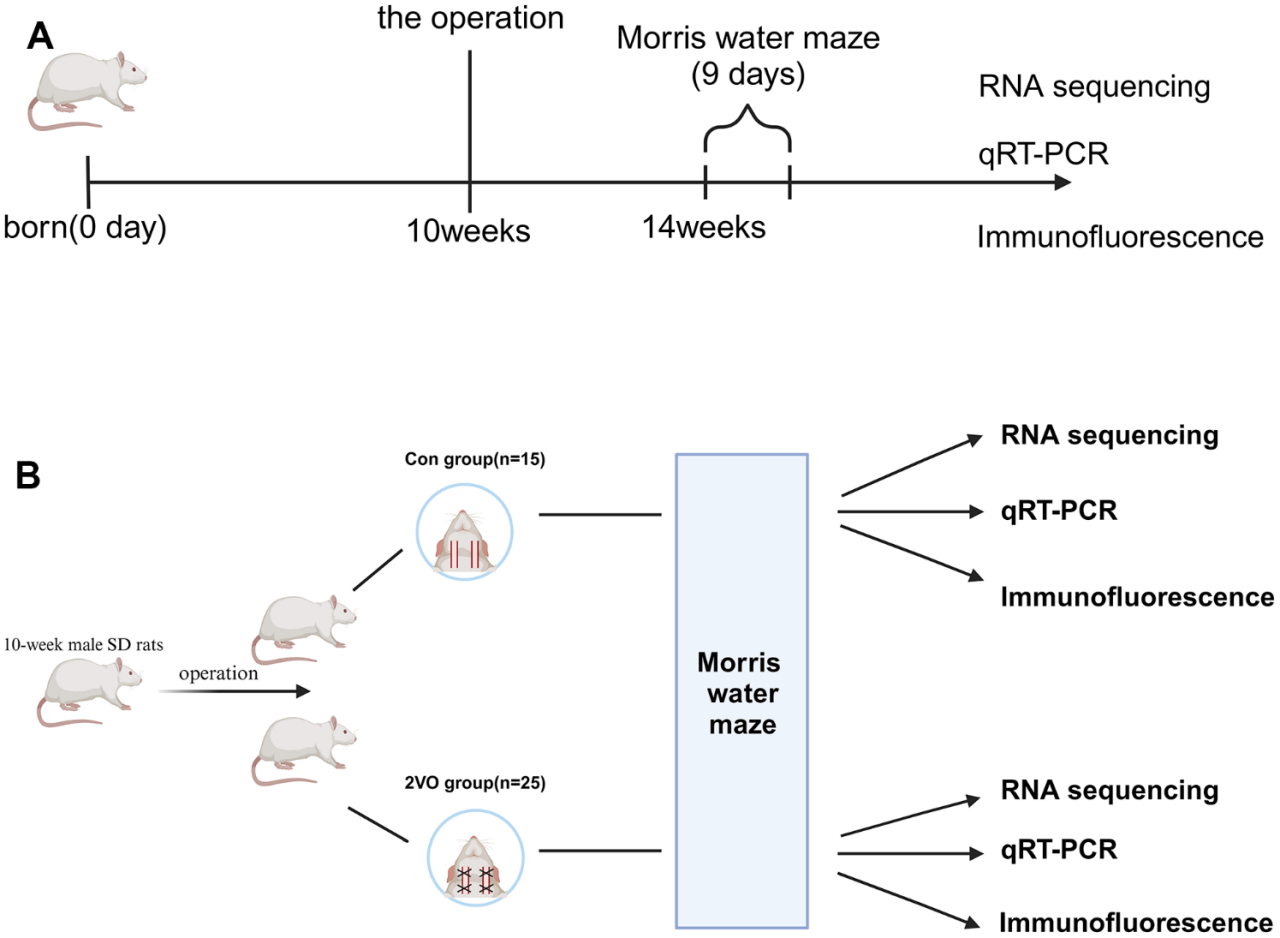
**Schematic representation of the experimental arrangement for two groups of rats.** (**A**) The timeline diagram of the experimental design; (**B**) The grouping and order of experimental arrangement. The Con group(n=15): the sham operation group, and the 2VO group(n=25): the bilateral common carotid arteries occlusion group.

40 10-week male SD rats were purchased from Hunan SJA Laboratory Animal Co., Ltd. The rats were housed in the SPF condition and accessed food and water ad libitum, with the 12-h light/dark cycle light on from 7:00 a.m. to 7:00 p.m. The rats were divided into two groups: the sham group (Con group, n=15), and the bilateral common carotid arteries occlusion group (2VO group, n=25) (Figure 1).

### Animal model of chronic cerebral hypoperfusion

The rats were anesthetized with 0.6% pelltobarbitalum natricum, and a median incision of the neck skin was performed. The bilateral sternocleidomastoid muscles were bluntly separated and the carotid sheath was exposed. The vagus nerve was dissociated and separated from the common carotid artery. At the site, 0.5 cm below the common carotid artery bifurcation, two 4-silks passed beneath the common carotid artery, and the common carotid artery blood flow was twice ligated and blocked (two blocking interval differences of 1cm). The contralateral common carotid artery was performed the same operation after a specific time interval. As mentioned in the introduction, after implementing this method, the success rate of modeling in rats increased to over 90% [15]. After the operation, the animals were placed on a 37° C thermostatic plate for anesthesia recovery and returned to the cage for further feeding. To verify the success of the 2VO model, a laser Doppler system was used to detect a 30% decrease in blood flow levels [16, 17].

### Morris water maze

After one month of chronic cerebral hypoperfusion, the Morris water maze was used to test the spatial cognitive function of rats. The rats were slowly placed into the water along the wall of the water maze and allowed to search for a platform 2cm above the water’s surface. During the process of learning to remember the location of the platform, the rats were allowed to swim freely within 60s, search and board the platform to remember the location information for 30s. If the platform position cannot be found within 60s, the rat will be guided to the platform to remember the position information for 30s. The water maze was divided into four quadrants, and the rats were trained in four quadrants for 7 days every day. After completing the training, the rats rested for a day. After removing the platform, the short-term memory of rats will be tested. During the learning and memory tests, the swimming speed, the latency to reach the platform, the retention time in the platform area, and the count of crossing in the platform position were recorded and used to analyze the learning and memory ability of rats.

### Immunofluorescence

After the rats were sacrificed by the pelltobarbitalum natricum (0.6%), the heart was rapidly perfused with 4 % paraformaldehyde. Then the brain tissue was removed from the skull and post-fixed in 4 % paraformaldehyde overnight. The rats’ brains were dehydrated twice in a 30 % sucrose PBS solution. After dehydration, the rats’ brains were cut into sections in cryostat microtome, and the sections were placed in antifreeze and stored at -20° C for use. After rinsing in PBS, the sections were treated with PBS containing 0.3 % TritonX-100 and blocked with PBS containing 5% donkey serum. Then the sections were incubated with primary antibody at 4° C for 24 hours. After rinsing, the sections were incubated with the species-specific secondary antibody at room temperature for 1 hour. At last, the sections were mounted with an anti-fluorescence quenching sealing agent containing DAPI and photographed under a fluorescence microscope, as well as subsequent quantitative analysis.

### The antibodies for immunofluorescence

The rabbit pAb NeuN antibody (Cat No.26975-1-AP) was purchased from Proteintech Group, Inc. (Wuhan, China). The rabbit pAb MAP2 antibody (GB11128-2), mouse mAb MBP antibody (GB12226), FITC conjugated Donkey Anti-Rabbit IgG (H+L)(GB22403) and Cy3 conjugated Donkey Anti-Mouse IgG (H+L) (GB21401) were purchased from Servicebio Technology Co., Ltd. (Wuhan, China). The rabbit pAb Iba1 (Cat No.019-19741) antibody was purchased from FUJIFILM Wako Pure Chemicals Corporation (Osaka, Japan). The goat pAb Iba1 antibody (NB100-1028) was purchased from Novus Biologicals, LLC (Centennial, CO, USA). The rabbit mAb synpasin I antibody (ab254349) was purchased from Abcam (Cambridge, UK). The mouse mAb postsynaptic density protein-95 (PSD-95) antibody (Cat No.75-028) was purchased from Neuromab (Davis, CA, USA). The fluorescent secondary antibodies conjugated Alexa Fluor® 594 AffiniPure F(ab’)_2_ Fragment Donkey Anti-Rabbit IgG (H+L)(Cat No.711-586-152), Alexa Fluor® 488 AffiniPure F(ab’)_2_ Fragment Donkey Anti-Goat IgG (H+L) (Cat No.705-546-147), Alexa Fluor® 488 AffiniPure F(ab’)_2_ Fragment Donkey Anti-Mouse IgG (H+L) (Cat No.715-546-151) were purchased from Jackson ImmunoResearch Laboratories, Inc (West Grove, PA, USA).

### RNA extraction and library construction

Total RNA was extracted using the mirVana miRNA Isolation Kit (Ambion) following the manufacturer’s protocol. RNA integrity was evaluated using the Agilent 2100 Bioanalyzer (Agilent Technologies, Santa Clara, CA, USA). The samples with RNA Integrity Number (RIN) ≥ 7 were subjected to the subsequent analysis. The libraries were constructed using TruSeq Stranded Total RNA with Ribo-Zero Gold according to the manufacturer’s instructions. Then these libraries were sequenced on the Illumina sequencing platform (HiSeqTM 2500), and 150 bp/125 bp paired-end reads were generated during high-throughput sequencing.

### RNA sequencing and differentially-expressed RNA (DE RNA) analysis

After removing the adapter, and filtering out low-quality bases and N-bases or low-quality reads, high-quality clean reads were gotten. Using hierarchical indexing for spliced alignment of transcripts 2 (HISAT2) [18] to align clean reads to the reference genome of the experimental species, the samples were assessed by genomic and gene alignment. The result of alignment with the reference genome was stored in a binary file. To generate a SAM file, we used Burrows-Wheeler Alignment tool (BWA) to align the sequencing reads of each sample with the reference genome [19]. Then CIRI [20] software was used to scan for PCC signals (paired chiastic clipping signals), and circRNA sequences were predicted based on junction reads and GT-AG cleavage signals. Using eXpress to make gene quantitative analysis, the fragments per kilobase million (FPKM) value and counts value (the number of reads for each gene in each sample) were obtained. The FPKM values, gene expression gene expression density in each sample, and the coefficient were used to evaluate the homogeneity of mRNA samples in each group. The reads per kilobase million (RPM) values, gene expression, and gene expression density in each sample and coefficient were used to evaluate the homogeneity of circRNA samples in each group. Using the DESeq (2012) R package [21] to normalize the counts, and using the nbinomTest function to calculate *p*-value and fold change values for the difference comparison. Differential transcripts were selected with *p*-values <= 0.05 and the fold change >= 1.5.

### RNA validation by qRT-PCR

qRT-PCR was employed to assess the data consistency between RNA sequencing and qRT-PCR. The detailed steps of RNA amplification by qRT-PCR were as previously [22]. Total RNAs from the 50 mg hippocampi tissue were extracted through the TRIzol Reagent (Invitrogen, Waltham, MA USA). The detailed procedure of qRT-PCR was as before. The relative expression of RNA levels was evaluated using the “ΔΔ Ct method” [23].

### GO function and KEGG pathway annotation analyses

Gene Ontology (GO) annotated the gene function in different terms. GO analysis displayed the annotated DE gene function in three levels: molecular function, biological process, and cellular component through a hypergeometric distribution test. Kyoto Encyclopedia of Genes and Genomes (KEGG) annotated the gene function in different pathways. KEGG analysis displayed the annotated DE gene function through a hypergeometric distribution test. For the hypergeometric distribution test, a P-value was obtained to determine the degree of the function and pathway enrichment of the DE genes. P-value < 0.05 was considered statistically significant [23].

### CircRNA-mRNA co-expression analyses

It was important for determining circRNAs gene regulation to study the level of co-expression between circRNA and mRNA. To analyze the co-expression level of circRNA and the gene, the expression levels of DE circRNA and genes were tested with the Pearson correlation analysis to calculate the correlation. A paired correlation coefficient > 0.8 and a P-value < 0.05 was considered as a threshold to determine the significant co-expression relationship [24].

### ceRNA construction of the circRNA-miRNA-mRNA network

The competing endogenous RNA (ceRNA) regulatory network was constructed using an intersection set of the predicted circRNA-miRNA interaction pairs and miRNA-mRNA interaction pairs. The detailed construction is as follows. miRanda program (v. 3.3a) [24] and the RNAhybrid database were searched to predict the circRNA-miRNA pairs [25]. miRanda score >150 and RNAhybrid energy<-25 were the thresholds to determine the interaction pairs between circRNAs and their targeted miRNAs. The miRBase database and the miRanda program (v. 3.3a) [24, 26] were used to predict miRNA-mRNA pairs. miRanda score >150 and RNAhybrid energy<-25 were the thresholds to determine the interaction pairs between circRNAs and their targeted miRNAs. Single residue pair match scores ≥ 150 and free energy of the double chains binding ≤ −30 kcal/mol along with strict 5’ seed pairing were the threshold to determine the interaction pairs between miRNAs and their targeted mRNAs. At last, the intersecting miRNA and the corresponding circRNAs and mRNAs were used to construct a ceRNA regulatory network of circRNA-miRNA-mRNA using Cytoscape software Cytoscape 3.6.1 [23, 27].

### Statistical analysis

Data were described as means ± standard error of the mean (SEM). Data analysis was carried out with SPSS 20.0 statistical software (SPSS Inc., Chicago, IL, USA). The repeated-measures analysis of variance was used to determine the statistical significance of RNAs among the three groups. The one-way analysis of variance, followed by Dunnett’s t-test, was used to determine the statistical differences and significance of the means between the two groups. P < 0.05 was considered a statistically significant difference of comparison. Fold changes (FCs) and P-value of tests were used to determine the statistical significance of the RNA sequencing data. FC ≥ 1.5 and P < 0.05 were used as thresholds for determining the differential expression of mRNAs and circRNAs [23].

## RESULTS

### CCH induced rats’ spatial learning and memory dysfunction

**Figure 2:**
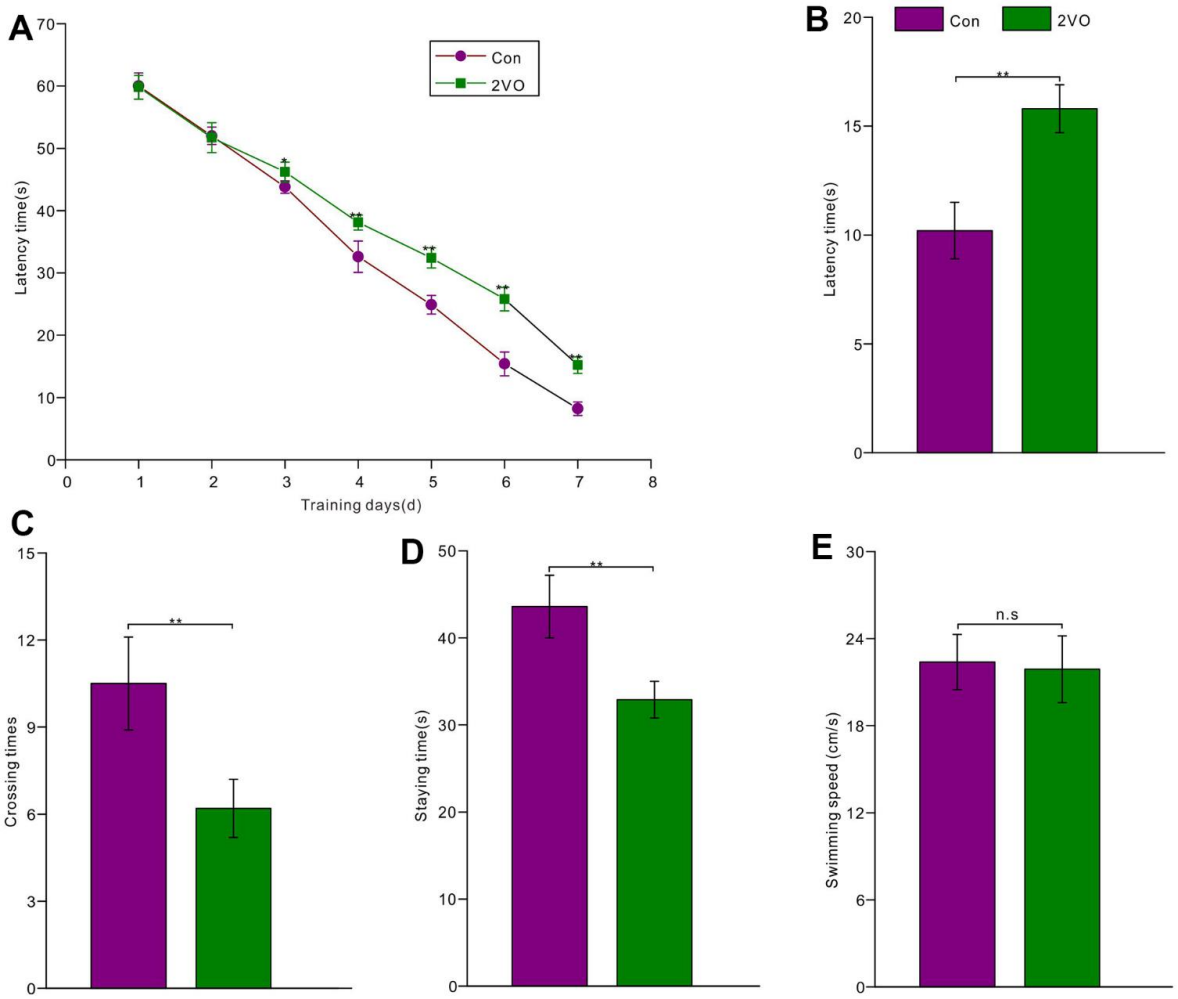
**The rats’ spatial learning and memory abilities were tested with the Morris water maze.** (**A**) The latency time was recorded during 7-day learning trials for the rats of the Con group (n=15) and 2VO group (n=25); After 1-day rest, the platform was removed and the rats were re-tested in the water maze. And (**B**) the latency time, (**C**) crossing times, (**D**) staying time, as well as (**E**) swimming speed was recorded and analyzed.

To investigate the effect of CCH on cognition, the Morris water maze was employed to test spatial learning and memory abilities among the two groups. The 7-day learning training trials showed that rats of the 2VO group had a longer latency time to reach the platform than those of the Con group (from 3^rd^ day to 7^th^ day *P*<0.05) (Figure 2A). After 1-day rest and removing the platform, the short-term memory was tested. The test data showed that the 2VO group had a longer latency time to reach the platform than the Con group (*P*<0.01) (Figure 2B). Meanwhile, the 2VO group had less crossing times to shuttle the platform area than the Con group (*P*<0.01) (Figure 2C). And the 2VO group had the less staying time in platform quadrant than these of the Con group (*P*<0.01) (Figure 2D). Nevertheless, there was no difference in swimming speed between the 2VO group and the Con group (*P*>0.05) (Figure 2E).

### CCH did not induce dramatic neuron loss in the brain

**Figure 3:**
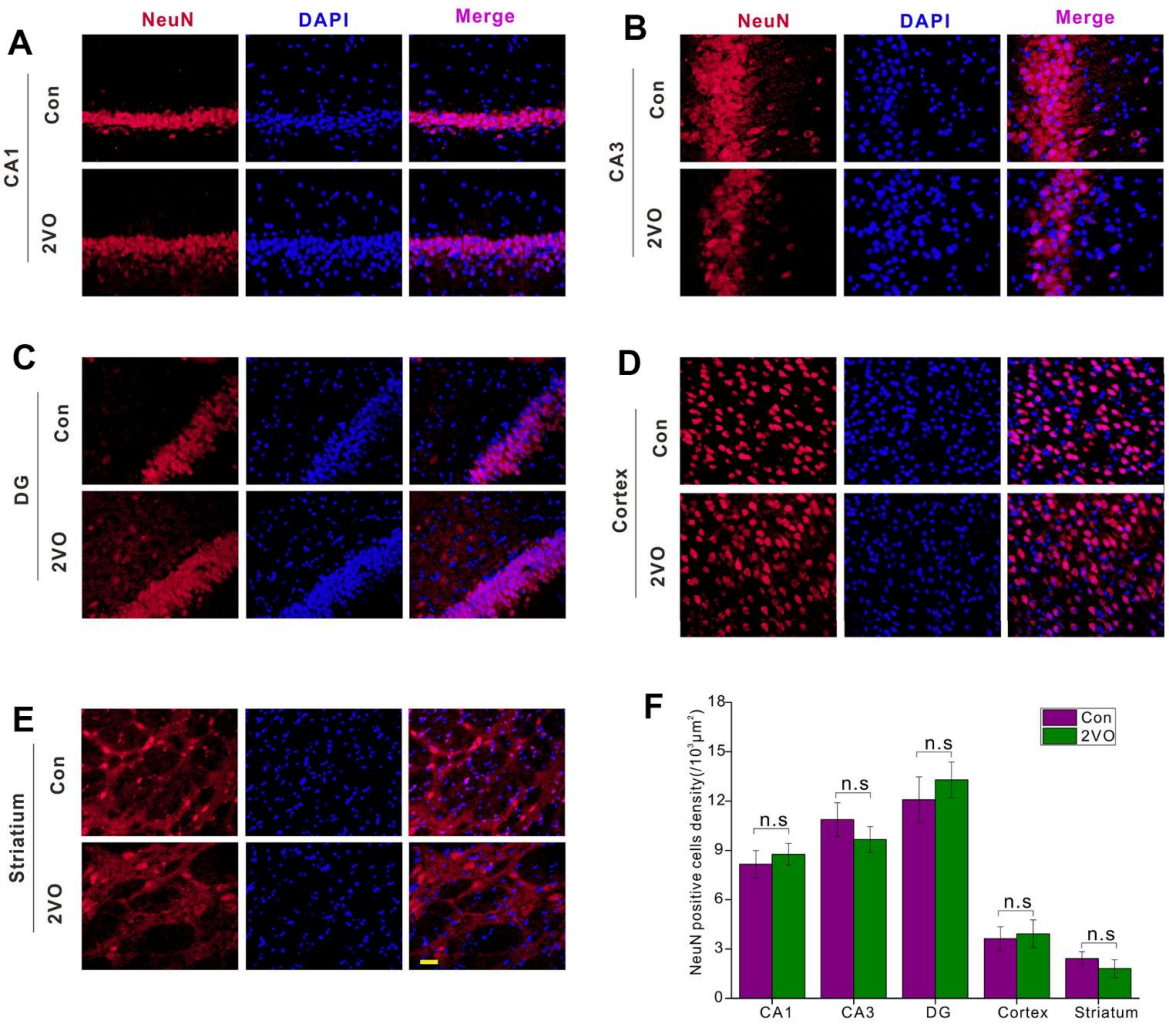
**The neuronal densities in different regions of the brain were evaluated with the immunofluorescent NeuN-labeled staining.** (**A**) CA1, (**B**) CA3, (**C**) DG, (**D**) Cortex, (**E**) Striatium regions were stained with NeuN antibody and DAPI. The red and blue staining indicated the NeuN-labeled neurons and the nucleus, respectively. The purple indicated the merge of both. (**F**) Count of NeuN-labeled neurons in different regions. Scale bar=10μm. [(Con group, n=3), (2VO group, n=3)].

To explore the mechanisms of cognitive dysfunction after CCH, the neuron density was examined by NeuN-staining. The NeuN-staining showed that there was no dramatic difference in the density of NeuN-positive neurons in CA1 (Figure 3A), CA3 (Figure 3B), DG (Figure 3C), cortex (Figure 3D), and striatum (Figure 3E) between the 2VO and Con group (*P*>0.05) (Figure 3F).

### CCH could reduce the neuronal fibers and aggravate demyelination in neurons of the hippocampus and cortex

**Figure 4:**
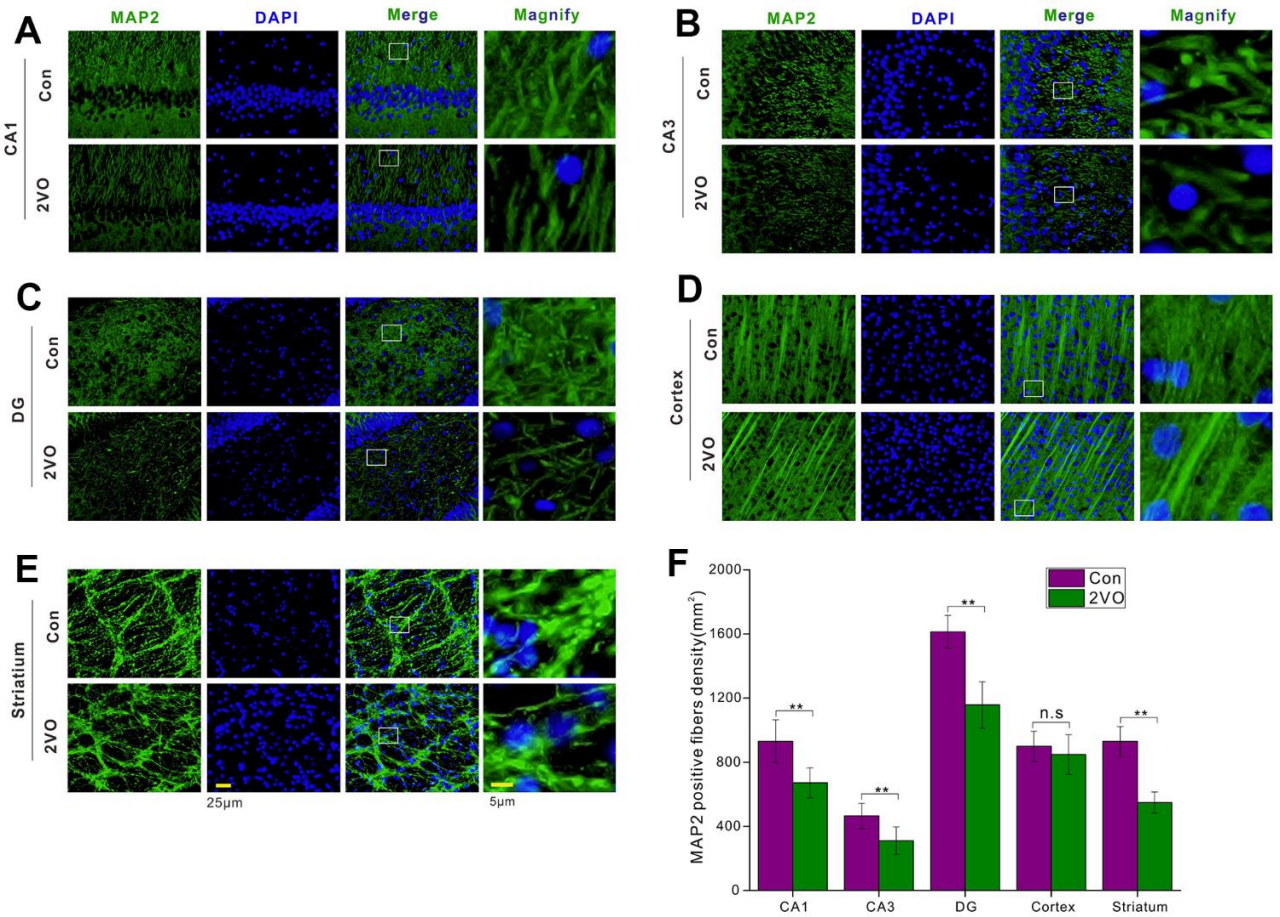
**The neuronal fiber density in different regions of the brain was evaluated with the immunofluorescent MAP2-labeled staining.** (**A**) CA1, (**B**) CA3, (**C**) DG, (**D**) Cortex, (**E**) Striatum regions were stained with MAP2 antibody and DAPI. The green and blue staining indicated the MAP2-labeled neuronal fibers and the nucleus, respectively. (**F**) The density of MAP2-labeled neuronal fibers in different regions. Scale bar = 25μm. The scale bar in the magnified image = 5μm. [(Con group, n=3), (2VO group, n=3)].

The nerve fibers were the important neuronal cables to connect the neurons in different regions and form the specific circuit, thereby certain brain functions could be played out. To figure out whether CCH could impact the nerve fiber, the brain slice was developed with MAP2 antibody. The MAP2 staining showed that CCH could reduce the density of MAP2-positive fiber in CA1 (Figure 4A), CA3 (Figure 4B), DG (Figure 4C), and striatum (Figure 4E), compared to the Con group (*P*<0.01) (Figure 4F). But there was no noticeable difference for MAP2-positive fiber between the 2VO group and the Con group (*P*>0.05) (Figure 4D, 4F).

**Figure 5:**
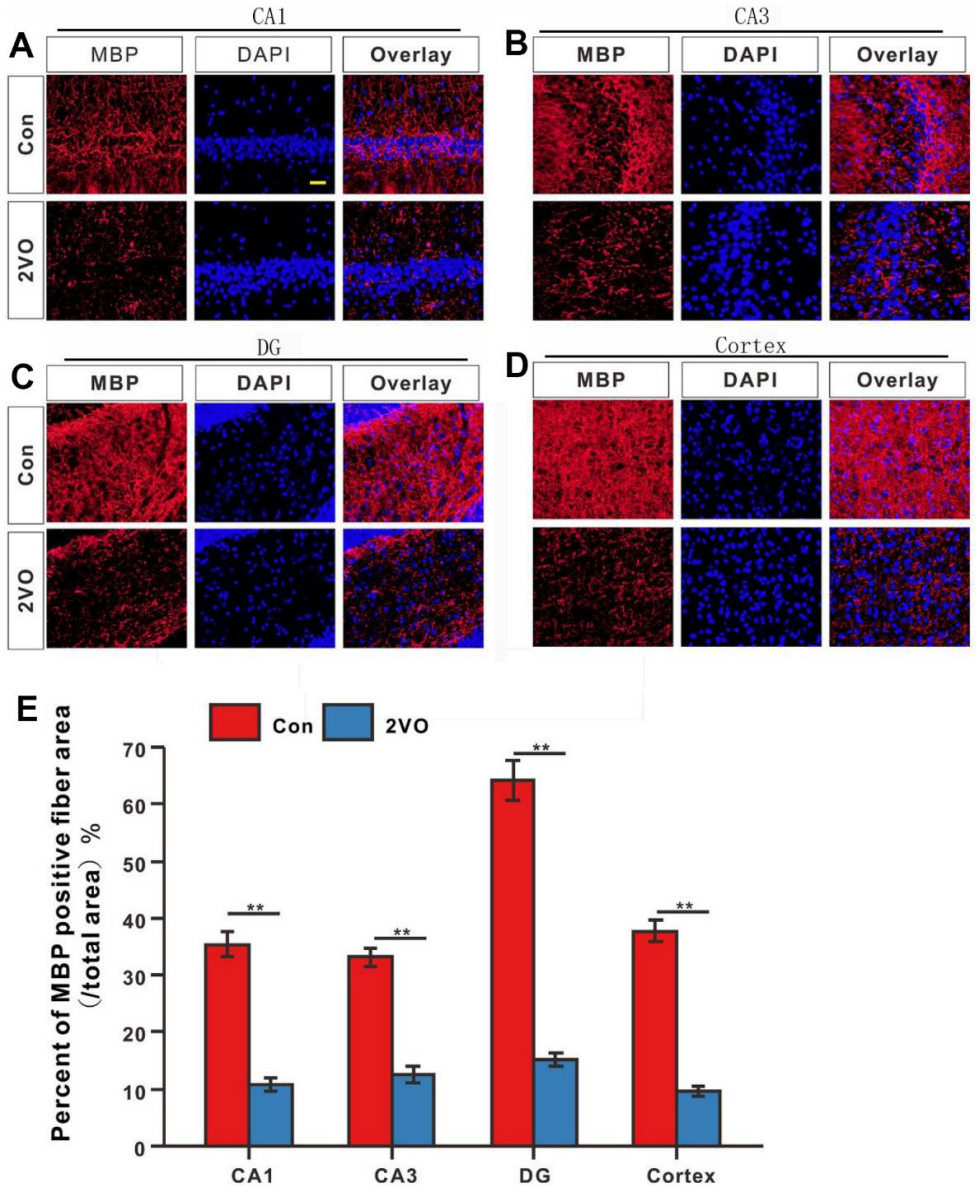
**The degree of myelination of neuronal fibers in different regions of the brain was evaluated with the immunofluorescent MBP-labeled staining.** (**A**) CA1, (**B**) CA3, (**C**) DG, and (**D**) Cortex regions were stained with MBP antibody and DAPI. The red and blue staining indicated the MBP-labeled neuronal fibers and the nucleus, respectively. (**E**) Percentage of the MBP-positive fiber area in various regions. Scale bar = 25μm. [(Con group, n=3), (2VO group, n=3)].

To further explore whether CCH induces the interactivity damage of neuronal axons, the MBP was detected with immunofluorescence. The MBP staining showed that the percent of MBP positive staining fiber area in CA1, CA3, DG, and cortex of 2VO (Figure 5A–5D), reduced dramatically than that in the Con group (*P*<0.01) (Figure 5E).

### CCH could activate and proliferate astrocyte and microglia of the hippocampus and cortex

**Figure 6:**
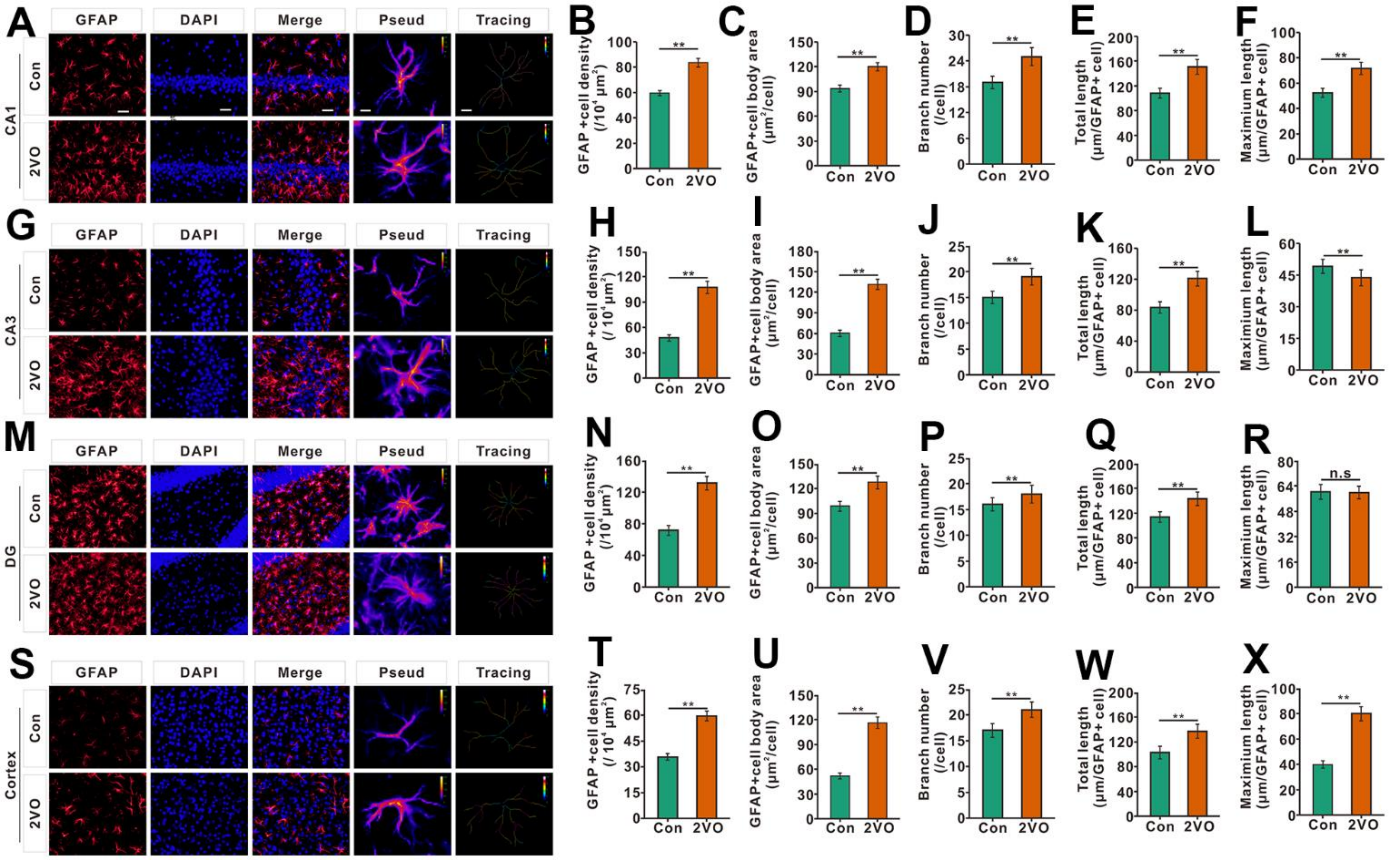
**The astrocyte profiles in different regions of the brain were evaluated with the immunofluorescent GFAP-labeled staining.** (**A**) astrocytes in CA1 were shown with GFAP antibody staining. (**B**) density, (**C**) body area, (**D**) branch number, (**E**) total projection length, and (**F**) maximum length of GFAP-positive cells were analyzed. (**G**) astrocytes in CA3 were shown with GFAP antibody staining. (**H**) density, (**I**) body area, (**J**) branch number, (**K**) total projection length, and (**L**) maximum length of GFAP-positive cells were analyzed. (**M**) astrocytes in the DG were shown with GFAP antibody staining. (**N**) density, (**O**) body area, (**P**) branch number, (**Q**) total projection length, and (**R**) maximum length of GFAP-positive cells were analyzed. (**S**) astrocytes in the cortex were shown with GFAP antibody staining. (**T**) density, (**U**) body area, (**V**) branch number, (**W**) total projection length, and (**X**) maximum length of GFAP-positive cells were analyzed. Scale bar=25μm. The scale bar in the magnified image=5μm. [(Con group, n=3), (2VO group, n=3)].

Neuroinflammation can be triggered by cerebral ischemia [28]. To explore whether CCH activates and proliferate astrocyte, the astrocytes were stained with glial fibrillary acidic protein (GFAP), a marker of astrocyte. The GFAP staining showed that the density of GFAP staining positive cells, the body area of GFAP staining positive cells, branch number, and total length of the project, in the CA1, CA3, DG, and cortex of the 2VO group, noticeably increased than that of the Con group (*P*<0.01) (Figure 6A–6E, 6G–6K, 6M–6Q, 6S–6W). In CA1 and cortex, the maximal length of projection in CA1 and cortex of 2VO, but not CA3 and DG, was remarkably greater than that of control group (*P*<0.01) (Figure 6F, 6L, 6R, 6X).

**Figure 7:**
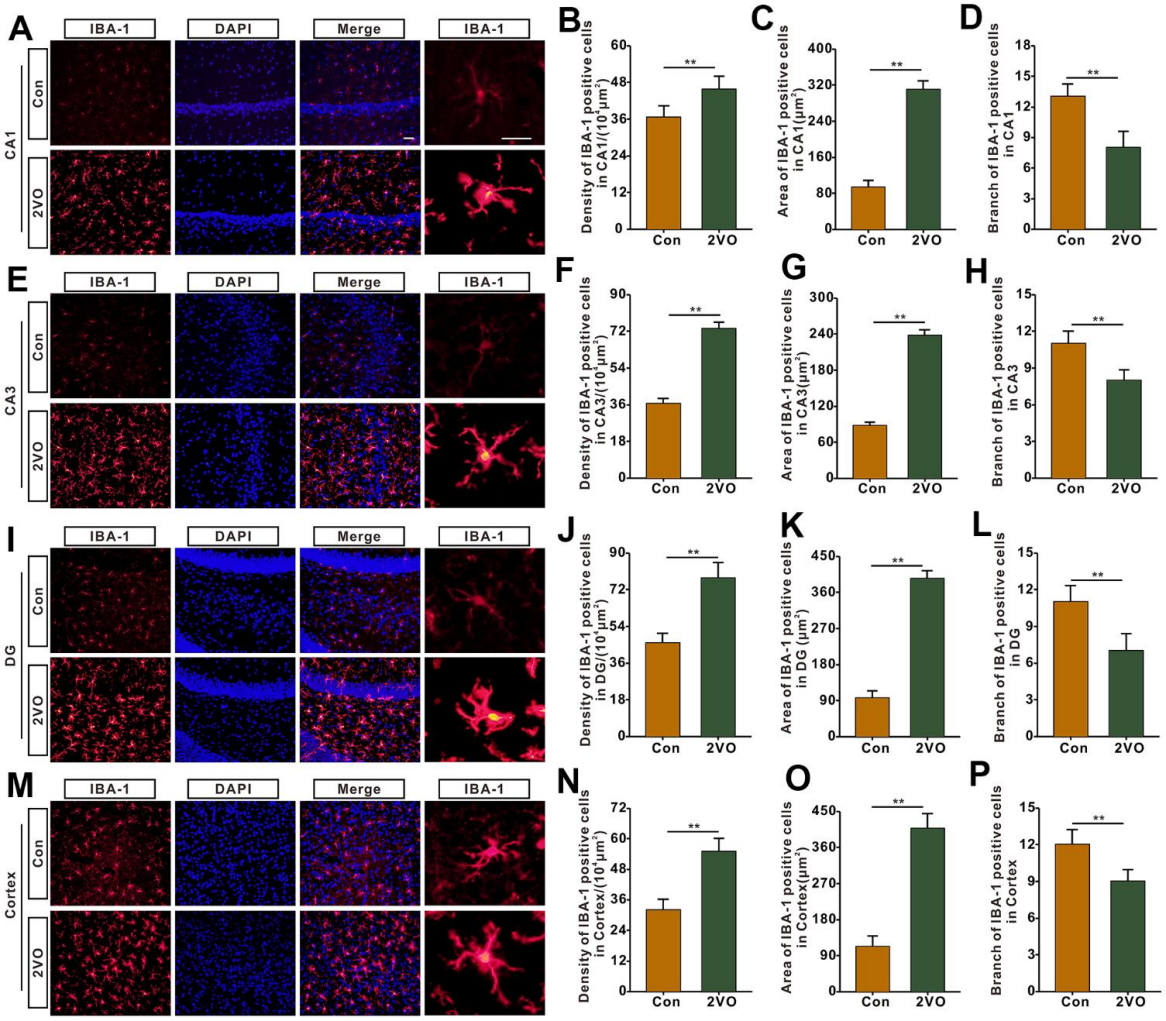
**The microglia profiles in different regions of the brain were evaluated with the immunofluorescent (IBA-1)-labeled staining.** (**A**) Microglia in CA1 were shown with IBA-1 antibody staining. (**B**) density, (**C**) body area and (**D**) branch number of IBA-1 positive cells were analyzed. (**E**) Microglia in CA3 were shown with IBA-1 antibody staining. (**F**) density, (**G**) body area and (**H**) branch number of IBA-1 positive cells were analyzed. (**I**) Microglia in DG were shown with IBA-1 antibody staining. (**J**) density, (**K**) body area and (**L**) branch number of IBA-1 positive cells were analyzed. (**M**) Microglia in the cortex were shown with IBA-1 antibody staining. (**N**) density, (**O**) body area and (**P**) branch number of IBA-1 positive cells were analyzed. Scale bar = 25μm. The scale bar in magnified image = 5μm. [(Con group, n=3), (2VO group, n= 3)].

Microglia is one of the main kinds of inflammation cells in the brain. To further explore whether CCH activates and proliferates microglia, the brain slices were stained with IBA-1, a marker of microglia. The IBA-1 staining showed that the density and the body area of IBA-1 staining positive cells in CA1, CA3, DG, and cortex of the 2VO group, markedly increased than that of the Con group (*P*<0.01) (Figure 7A–7C, 7E–7G, 7I–7K, 7M–7O). And branch number of the CA1, CA3, DG, and hippocampus, was remarkably less than that of the Con group (P<0.01) (Figure 7D, 7H, 7L, 7P).

### CCH induces the loss of synapses and increased phagocytosis of synapses by microglia

**Figure 8:**
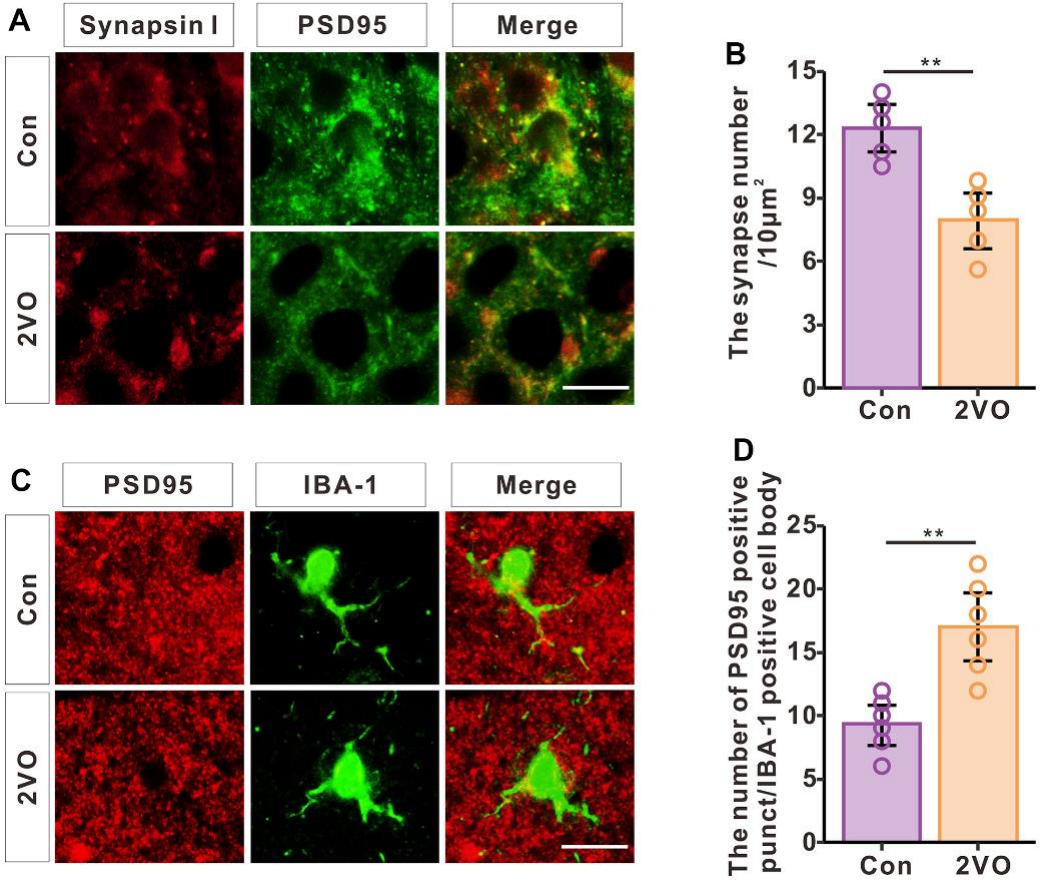
**CCH induces the loss of synapses and increased phagocytosis of synapses by microglia.** (**A**) Brain slices of the hippocampus were stained with synapsin and PSD95 antibodies to show the synapses. (**B**) The density of synapses was analyzed (n=5). (**C**) Brain slices of the hippocampus were stained with PSD-95 and IBA-1 antibodies to show the engulfing of synapses by microglia. (**D**) The PSD-95 positive punctation number of microglia’s cell body was analyzed (n=6). Scale bar = 2.5μm.

To investigate synapse levels after CCH, the brain slice was stained with synapsin I and PSD-95 (Figure 8A). The immunofluorescence data showed that CCH dramatically decreased the density of synapses (P<0.01) (Figure 8B). To further explore whether the synapse loss was induced by phagocytosis of synapses from microglia, the brain slice was stained with PSD-95 and IBA-1 (Figure 8C). The immunofluorescence data showed that CCH dramatically increased the PSD-95 positive punct number of microglia’ cell body (P<0.01) (Figure 8D). These suggested that CCH activate the microglia to engulf the PSD-95 to contribute to the synapse loss.

### Homogeneity analysis of RNA sequencing

**Figure 9:**
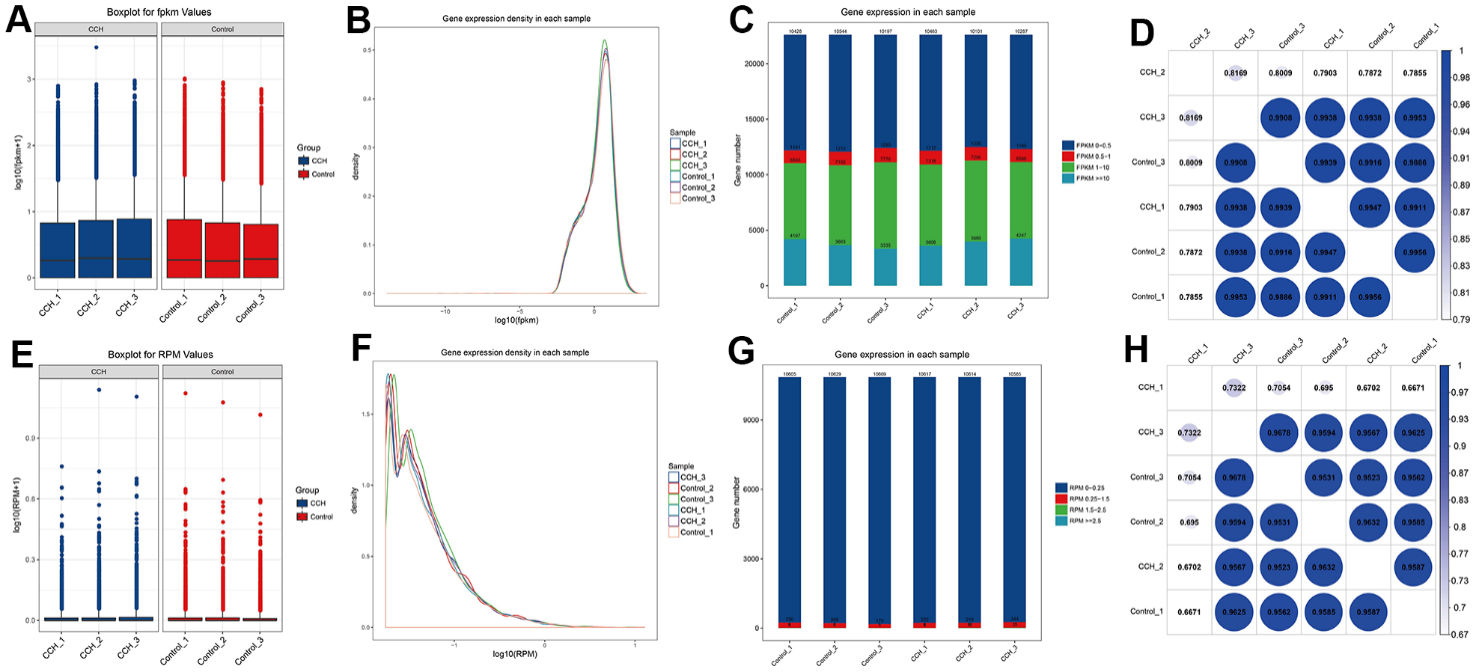
**The homogeneous quality analysis of RNA sequencing in different samples.** (**A**) log_10_(FPKM) level in different groups; (**B**) Gene expression density of FPKM bases in each sample; (**C**) The gene number of different intervals of FPKM bases values(0-0.5, 0.5-1, 1-10, >=10); (**D**) Coefficient analysis of gene number of FPKM bases in different groups; (**E**) log_10_(RPM) level in different groups; (**F**) Gene expression density of RPM bases in each sample; (**G**) The gene number of different intervals of RPM bases values(0-0.25, 0.25-1.5, 1.5-2.5, >=2.5); (**H**) Coefficient analysis of gene number of RPM bases in different groups. [(Con group, n=3), (2VO group, n=3)].

To determine whether the data from RNA sequencing of the different groups had less difference, FPKM bases and RPM bases were analyzed. The data showed that the absolute FPKM value (log_10_(PFKM)) and RPM value (log_10_(RPM)) in the CCH and Con groups had no noticeable difference (Figure 9A, 9E). To further investigate the distribution of FPKM and RPM, both the density of log_10_(FPKM) and log_10_(RPM) had a highly consistent distribution (Figure 9B, 9F). Moreover, the gene numbers in different FPKM and RPM values were almost near (Figure 9C, 9G). Besides, the correlation analysis showed that FPKM and RMP values in different groups were highly correlated (Figures 9D, 8H). These suggested that the sequencing data from the different groups in the CCH and Con groups had high homogeneity suitable to be further analyzed.

### Profile of circRNA and mRNA expression in rats’ hippocampus after CCH

Through RNA sequencing, 10850 circRNAs were identified and the number of upregulated and downregulated circRNAs respectively were 48 and 30(|log2(FC)|>0.56, *P*<0.05). 18846 mRNA were identified and the number of upregulated and downregulated mRNAs respectively were 34 and 25(|log2(FC)|>0.56, *P*<0.05).

**Figure 10:**
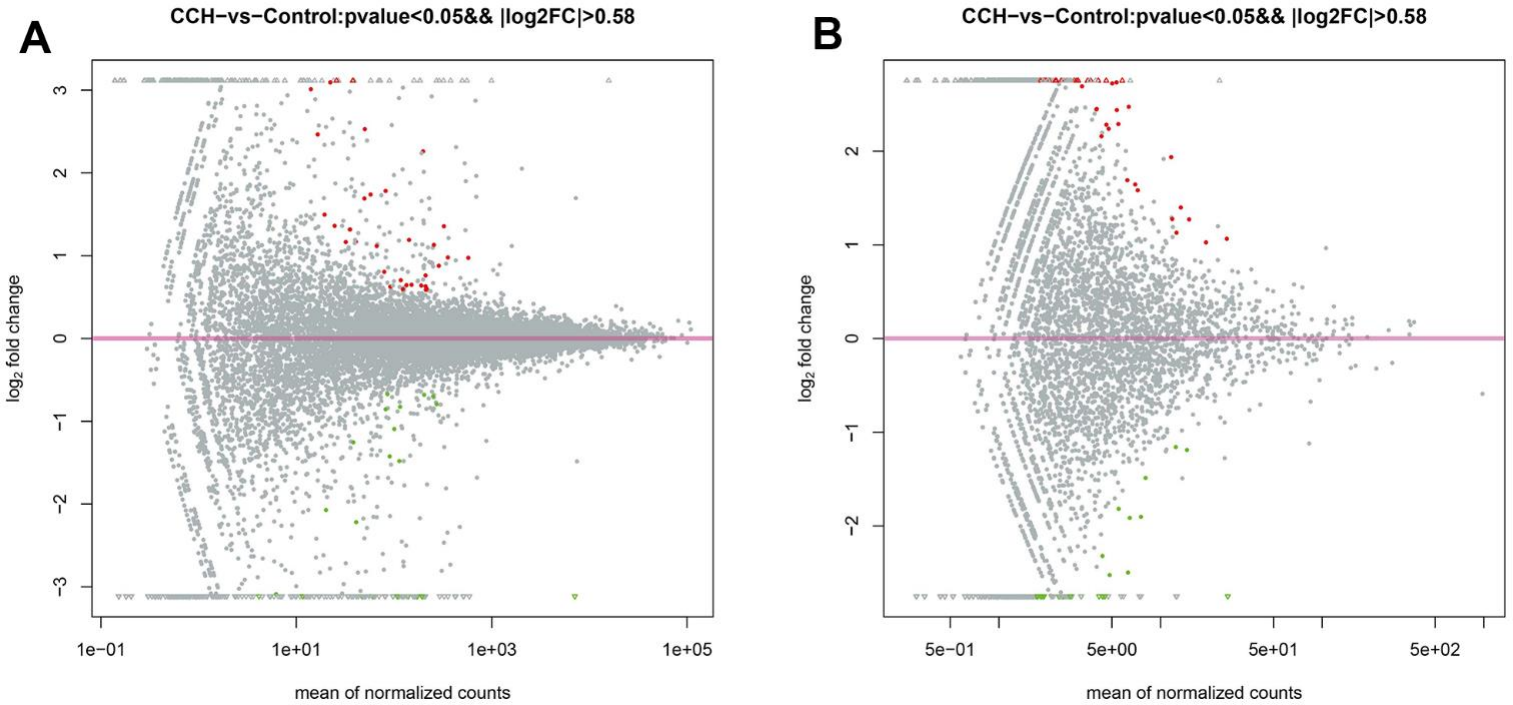
**The MA plot of RNA sequencing data.** (**A**) The MA plot of mRNA data; (**B**) The MA plot of circRNA data. The Y-axis indicates the log_2_(fold change), and the X-axis indicates the mean of normalized counts. The pink horizontal line represents the boundary between increased and decreased gene expression. [(Con group, n=3), (2VO group, n=3)].

The top 5 upregulated mRNAs were LOC100912642, LOC108348080, C4a, Katnal1, and LOC103689941, respectively with fold changes of 44.59, 34.01, 8.54, 8.07, and 5.77, compared to the control group. The top 5 downregulated mRNAs were Tph1, LOC100912599, LOC100910575, Lilrb3l, and LOC103689983, respectively with fold changes of 0.00073, 0.031, 0.031, 0.042, and 0.048, compared to the Con group. The top 5 upregulated circRNAs were circRNA_09183|Chr7:112745541_112761127_-, circRNA_04107|Chr17:81990547_82003698_+, circRNA_09363|Chr8:508994 519112 +, circRNA_05625|Chr2:257936685 257945564_+, circRNA_06465|Chr3:129077671_129078456_-, respectively with fold change of 9.01, 8.48, 8.39, 7.87, and 6.98, compared to control group. The top 5 downregulated circRNAs were Tph1, LOC100912599, LOC100910575, Lilrb3l, and LOC103689983, respectively with fold changes of 0.10, 0.17, 0.17, 0.19, and 0.26 compared to the control group. The MA plot was constructed to visualize the dramatically different expression levels of circRNA and mRNAs in the CCH and the Con groups (Figure 10A, 10B).

### Validation of RNA sequencing by qRT-PCR

**Figure 11:**
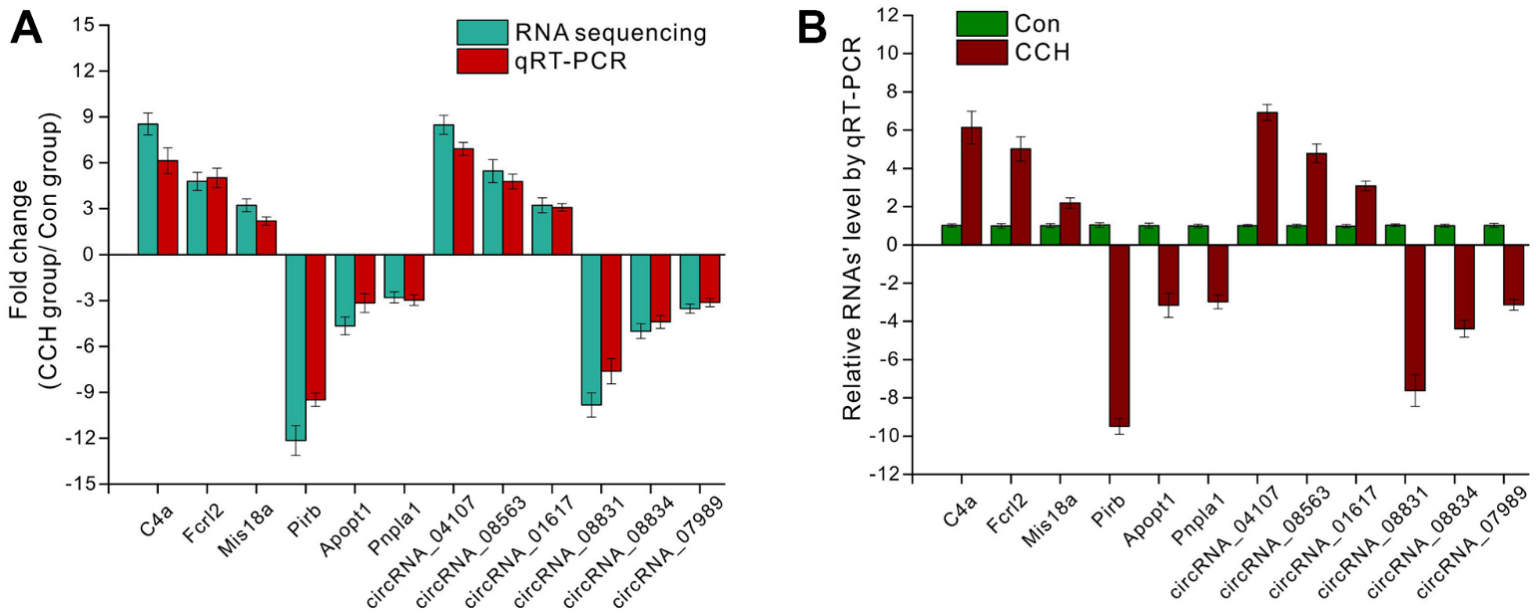
**The validation of RNA sequencing by qRT-PCR.** (**A**) The fold change of selected mRNA and circRNA detected by RNA sequencing and qRT-PCR; (**B**) Relative mRNA level by qRT-PCR. [(Con group, n=3), (2VO group, n=3)].

To validate the RNA sequencing data, qRT-PCR was employed to compare the consistency between the RNA sequencing and qRT-PCR. 3 upregulated mRNAs (C4a, Fcrl2, Mis18a) and circRNAs (circRNA_04107, circRNA_08563, circRNA_01617) and downregulated mRNAs (Pirb, Apopt1, Pnpla1) and circRNAs (circRNA_08831, circRNA_08834, circRNA_07989) were selected to be verified. The data showed that there were similar trends for gene expressions between sequencing and qRT-PCR (Figure 11A). For the selected upregulated mRNAs and circRNAs in CCH groups, the qRT-PCR data showed that these genes increased compared to the Con groups. Similarly, qRT-PCR data showed that these downregulated genes decreased compared to the Con groups (Figure 11B). These suggested that RNA sequencing had enough validation for further analysis.

### GO and KEGG enrichment analysis of DE mRNAs

**Figure 12:**
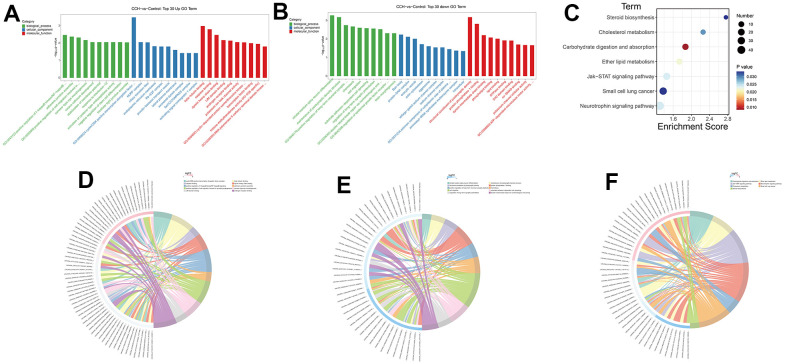
**GO and KEGG enrichment analysis of DE mRNAs.** Through a hypergeometric algorithm, the DE mRNAs were enriched on different GO terms and KEGG pathways. (**A**) Top 30 GO terms with upregulated DE mRNAs. (**B**) Top 30 GO terms with downregulated DE mRNAs. (**C**) enriched KEGG pathways. (**D**) The chord diagram showed the corresponding relationship between upregulated GO enrichment terms and related DE circRNAs. (**E**) The chord diagram showed the corresponding relationship between downregulated GO enrichment terms and related DE circRNAs. (**F**) The chord diagram showed the corresponding relationship between KEGG pathways and related DE circRNAs. [(Con group, n=3), (2VO group, n=3)].

To analyze the probable mechanisms underlying the altered neuropathology and cognitive function, the RNA sequencing data were enriched on different GO terms and KEGG pathways. The GO enrichment showed that the top 5 upregulated enrichment terms in the biological process were positive regulation of I-kappaB kinase/NF-kappaB signaling, adherens junctions assembly, positive regulation of cell migration involved in sprouting angiogenesis, camera-type eye morphogenesis, corpus callosum development. The top 5 upregulated enrichment terms in cellular components were cyclin-dependent kinases/CDK, positive transcription elongation factor complex, NURF complex, ciliary transition fiber, endoplasmic reticulum membrane, and Rb-E2F complex. The top 5 upregulated enrichment terms in molecular function were beta-tubulin binding, caspase binding, dynein heavy chain binding, LIM domain binding, and androgen receptor binding (Figure 12A). The GO enrichment showed that the top 5 downregulated enrichment terms in the biological process were striatal medium spiny neuron differentiation, maintenance of postsynaptic density structure, positive regulation of long-term neuronal synaptic plasticity, chemotaxis, cell migration. The top 5 downregulated enrichment terms in cellular components were Barr body, protein-DNA complex, exocytic vesicle, and actin cytoskeleton aggresome. The top 5 downregulated enrichment terms in molecular function were structural constituent of the postsynaptic density, protein phosphatase 1 binding, dynein intermediate chain binding, phospholipid binding, and lipid binding (Figure 12B). The top 5 enrichment KEGG pathways were Steroid biosynthesis, Cholesterol metabolism, Carbohydrate digestion and absorption, Ether lipid metabolism, Jak-STAT signaling pathway (Figure 12C). The corresponding relationship between enrichment terms and related DE circRNAs was demonstrated in the chord diagram (Figure 12D, 12E, 12F). In these enriched GO terms and KEGG pathways, the immunology, inflammation, and metabolisms change-related aspects were the main enrichment subjects. These suggested that the immunology, inflammation, and metabolism dysfunction signaling pathway took part in cognitive dysfunction after CCH and circRNAs might participate in regulating the expression of DE mRNAs.

### Co-expression network analysis

**Figure 13:**
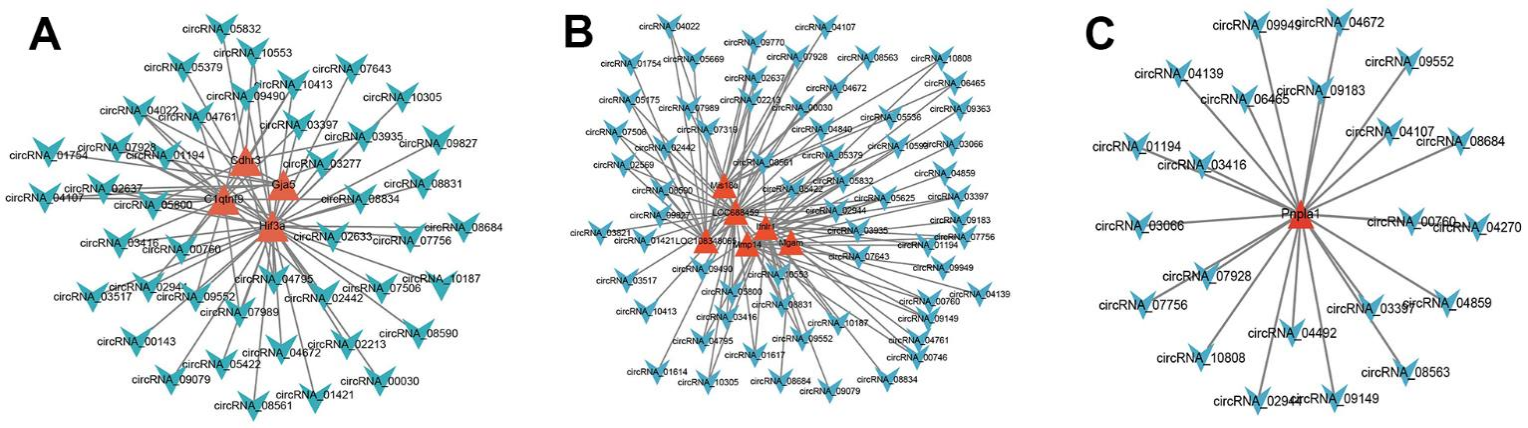
**Co-expression network analysis.** According to the threshold of P<0.05, Pearson correlation coefficient >0.85, the circRNA and mRNA pairs were filtered to construct the co-expression network. (**A**–**C**), The red triangle represented the mRNAs, and the Azure arrow represented circRNAs. [(Con group, n=3), (2VO group, n=3)].

Through Pearson correlation analysis between circRNA and mRNA expressions, the paired circRNA and mRNA met the threshold were used to construct the co-expression network. The representative co-expression net was demonstrated (Figure 13A, 13B). The same one gene was co-expressed with many circRNAs. These suggested that DE mRNAs may be regulated by many circRNAs.

### Ternary ceRNA network analysis

**Figure 14:**
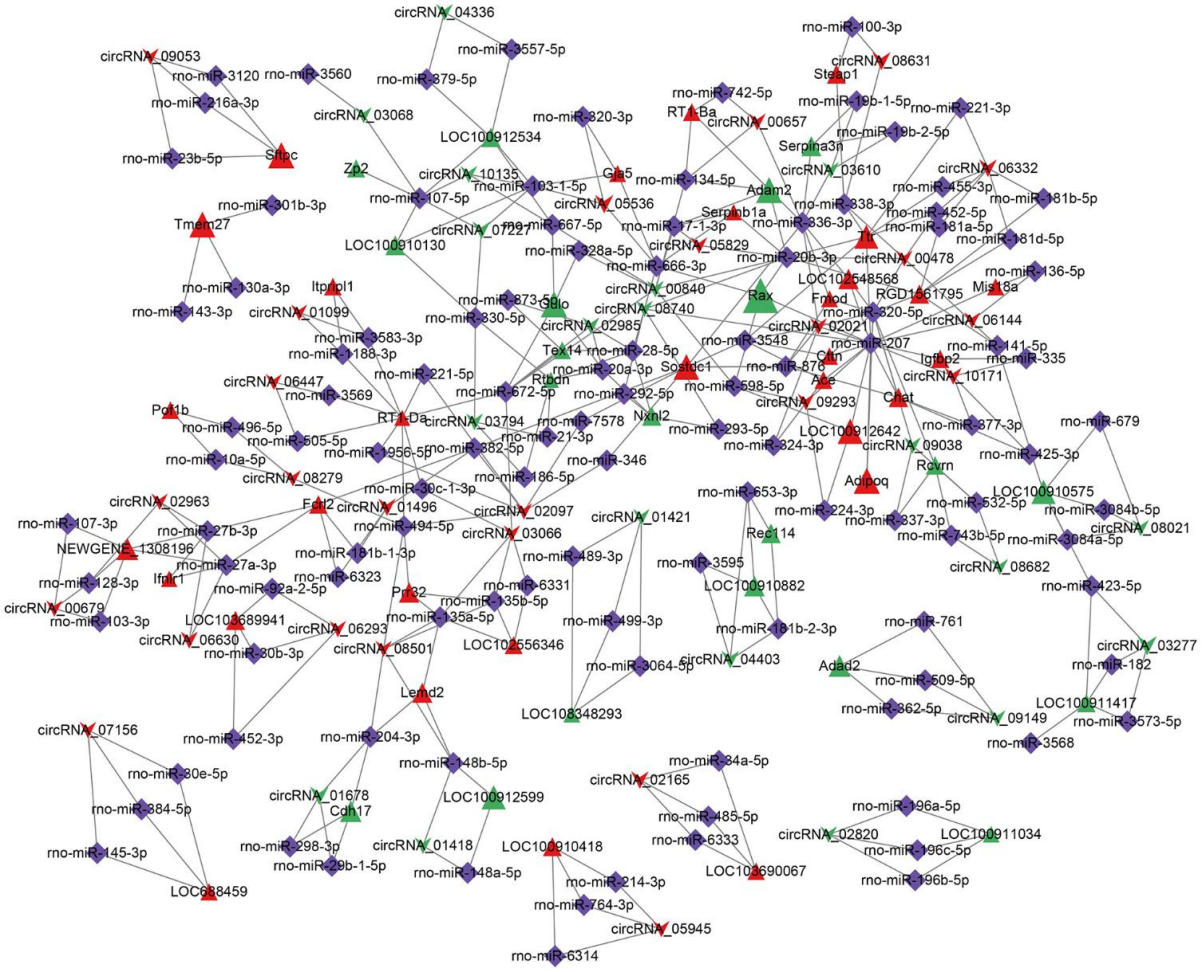
**CircRNA-miRNA-mRNA ceRNA network.** The top 48 MuTATE score pairs were used to plot a ternary network diagram. The arrow represented circRNA, the triangle represented mRNA, diamond represented miRNA. Red and green colors represented upregulation or downregulation. The gray line represents the regulation effect. [(Con group, n=3), (2VO group, n=3)].

Through prediction analysis, the 44665 miRNA-mRNA pairs and 4675 miRNA-circRNA pairs were predicted. The common miRNAs in two pair kinds were the nodes to construct the ceRNA network based on the MuTATE score. The top 48 MuTATE score pairs were used to plot a ternary network diagram (Figure 14). The network plot showed that the DG mRNAs were regulated by different circRNAs through miRNAs.

### GO and KEGG analysis of the mRNAs in the ceRNA network

**Figure 15:**
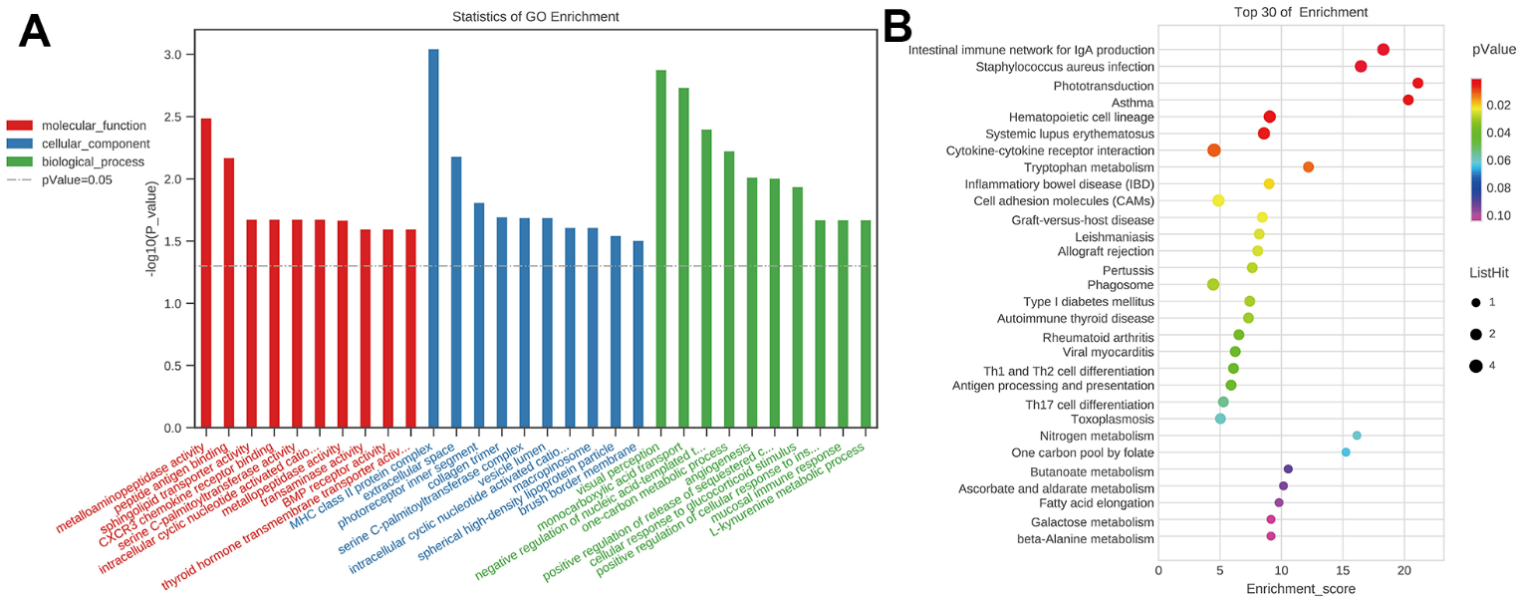
**GO and KEGG analysis of the mRNAs in ceRNA network.** (**A**) GO analysis, p-Value=0.05 as the threshold of significant enrichment. Red represented molecular function, blue represented cellular component, green represented biological process, the x-axis represented different terms, y-axis –log10(P-value); (**B**) KEGG analysis, the x-axis represented enrichment score, y-axis represented different pathways. [(Con group, n=3), (2VO group, n=3)].

To investigate the probable roles of mRNAs of ceRNA, the GO and KEGG analyses were employed. The GO enrichment showed that the top 5 enrichment terms in the biological process were visual perception, monocarboxylic acid transport, negative regulation of nucleic acid-templated transcription, one-carbon metabolic process, and angiogenesis. The top 5 enrichment terms in cellular components were major histocompatibility complex (MHC) class II protein complex, extracellular space, photoreceptor inner segment, collagen trimer, and serine C-palmitoyltransferase complex. The top 5 enrichment terms in molecular function were metalloaminopeptidase activity, peptide antigen binding, sphingolipid transporter activity, CXCR3 chemokine receptor binding, serine C-palmitoyltransferase activity (Figure 15A). The KEGG pathway enrichment showed that top 5 enrichment pathways were Phototransduction, Asthma, Intestinal immune network for IgA production, *Staphylococcus aureus* infection, Tryptophan metabolism (Figure 15B). GO terms and KEGG pathways data in mRNAs of the ceRNA network showed that immunology and inflammation related terms and pathway had highly enriched. These also suggest that there were lots of probable circRNAs involved in regulating mRNAs related to immunology and inflammation to participate in cognitive dysfunction after CCH.

## DISCUSSION

In the present study, cognitive dysfunction after CCH was further investigated and the underlying neuropathological alterations were observed. The number of neurons in the brain did not decrease. The neuronal fibers were markedly reduced after CCH by MAP2 staining, and the demyelination of neuronal fibers was aggravated by MBP staining. Meanwhile, the neuroinflammation deteriorated after CCH. The astrocytes and microglia proliferated significantly in the hippocampus, and the activated state of astrocytes and microglia also dramatically increased. Meanwhile, synapse loss was induced by CCH, and the engulfment of the synapse by microglia increased. With GO and KEGG enrichment analysis, the immunological and inflammation terms and pathways were significantly enriched with the DE mRNA. Meanwhile, the DE circRNAs had a remarkable coexpression relationship with DE mRNA. Through the binding prediction of DE circRNAs and mRNAs with miRNA, the ternary ceRNA network was constructed. For the DE mRNA in the ternary ceRNA network of circRNA-miRNA-mRNA subjects, GO terms and KEGG pathways enrichment analysis showed the immunological and inflammation-related terms and pathways were significantly enriched.

CCH could induce cognitive dysfunction in many diseases, including vascular dementia [29], Alzheimer’s disease [30, 31], and Parkinson’s dementia [32]. Meanwhile, emotional dysfunction also can be induced by CCH [33–35]. The present study showed that CCH induced the rats’ spatial cognitive dysfunction and re-identified the CCH’s injury on cognition. In future studies, more behavioral paradigms should be designed or employed to investigate the cognitive domain to deeply uncover the CCH’s effect on cognition. CCH reduces the blood supply to the brain and the metabolism of glucose and oxygen decreases, which contributes the oxidative stress [36], neuroinflammation [29, 37], Aβ accumulation and tau hyperphosphorylation [38], white matter lesion [39], blood-brain barrier disruption [30] and synaptic injury and dysfunction [40, 41]. The integrity of neural fiber injuries and demyelination are very important to maintain normal cognitive function. The inhibitor of Na+/H+ exchanger-1 (NHE1) could inhibit demyelination and axonal damage in white matter tracts and the hippocampus, and significantly improve cognitive performance after CCH [37]. Deletion of C3ar1 significantly inhibited aberrant microglial activation to phagocytose myelin and prevented white matter injury after hypoperfusion [39]. A colony-stimulating factor 1 receptor inhibitor, PLX3397, was employed to deplete microglia, which can suppress white matter injury, and reduce the expression of interleukin 6 and tumor necrosis factor -α in the CCH mice model [42]. The present study also showed CCH induced neural fiber injuries and demyelination. Moreover, the proliferation and activation of inflammation cells including astrocytes and microglia were discovered, and the microglia engulfing the synapses was observed. These suggested that inflammation cells in CNS may mediate the neural fiber injuries to induce cognitive dysfunction after CCH. Besides, activated astrocytes and microglia can generate and release lots of inflammatory mediators and ROS, which can directly impair neuronal function and damage neuronal morphological structure. Through suppressing HMGB1, the levels of TNF-α, IL-1β, and IL-6 decreased, and hippocampal atrophy and cognitive decline were attenuated in the CCH model of mice [43]. Maresin 1 and β-hydroxybutyrate improve cognitive decline by inhibiting inflammation after CCH [44, 45]. Hence, the neuroinflammation induced by astrocytes and microglia plays a critical role in cognitive dysfunction after CCH.

Many factors could regulate the expression of neuroinflammatory factors, such as endogenous inflammatory mediators [46], transcription factors [15], miRNAs [47], and so on. Among these factors, miRNAs were important players participating in regulating the expression of neuroinflammatory factors after CCH. MiRNA-195 could prevent microglia activation by regulating CX3CR1-related signaling pathways [47]. MiR-322-5p could attenuate neuroinflammation and cognitive impairment in the CCH rat model through regulating negatively regulate tetraspanin 5 (TSPAN5) [48]. MiRNA-15a/16-1 knockout could prevent neuropathological impairment, neuroinflammation, and cognitive dysfunction after CCH [49]. These also suggest that miRNAs play critical roles in regulating neuroinflammation after CCH. In the present study, many DE genes by RNA sequencing were enriched on the inflammation-related terms and pathway, which further meant that neuroinflammation plays an indispensable role in neuropathological damage and cognitive dysfunction after CCH. The circRNA is an important regulatory small RNA, which can function as miRNAs’ sponge molecules by competing for the binding of mRNA with miRNAs [50, 51]. ciRS-7 is a well-characterized circRNA, having more than 70 binding sites with miR-7, and distributed in many tissues, especially the brain, which regulates many known and unknown cellular events [52]. Many other circRNAs, such as circHIPK3 and circBIRC6 [53], could bind to more than one miRNA. Hence, in some state, the complex competing endogenous circRNA-miRNA-mRNA network exists to comprehensively regulate the gene expression within tissues. CircRNA_09505 aggravated inflammation through the miR-6089/AKT1/NF-κB signaling pathway [54]. Circular RNA Cdyl promoted inflammation by fostering M1 macrophage polarization [55]. CircRNA_103765 acted as a proinflammatory factor by sponging miR-30 [56]. In the present study, 78 DE circRNAs 59 and DE mRNAs were identified. Through the prediction of miRNA binding with circRNAs and mRNAs, the common miRNAs were used as link crosses to construct the ternary interaction network. Lots of circRNA-miRNA-mRNA were predicted and the effect of mRNAs was involved in neuroinflammation through GO and KEGG enrichment analysis. These suggested that circRNA plays an important role in regulating neuroinflammation in the brain after CCH, which eventually results in neuropathological damage and cognitive dysfunction after CCH. However, in the present study, the miRNAs were not detected, and the entire link network was based solely on structure prediction, which introduces a certain degree of uncertainty. Moreover, the effects of circRNAs on neuroinflammation have not been verified in a reliable experimental design. In future studies, verification experiments should be carried out to identify and screen the critical circRNAs involved in regulating neuroinflammation after CCH. This may supply the potential targets against cognitive dysfunction after CCH.

In conclusion, the present study found that neuroimmune dysfunction and neuroinflammation may play an important role in neuropathological changes and cognitive dysfunction after CCH, in which circRNA possibly participates. Hence, circRNA might be an important target to counter the neuropathological changes and cognitive dysfunction after CCH.
